# A highly versatile and easily configurable system for plant electrophysiology

**DOI:** 10.1016/j.mex.2016.05.007

**Published:** 2016-05-25

**Authors:** Benet Gunsé, Charlotte Poschenrieder, Simone Rankl, Peter Schröeder, Ana Rodrigo-Moreno, Juan Barceló

**Affiliations:** a Lab. Fisiología Vegetal, Facultad Biociencias, Universidad Autónoma de Barcelona, E-08193 Bellaterra, Spain; b Helmholtz Zentrum München, Deutsches Forschungszentrum für Gesundheit und Umwelt (GmbH), Ingolstädter Landstr. 1, 85764 Neuherberg, Germany; c Department of Plant, Soil and Environmental Science, Polo Scientifico, Viale delle Idee 30, 50019 SestoFiorentino, Firenze, Italy

**Keywords:** Improved system for plant electrophysiology, Plant root, Membrane potential, Ion flux measurement, Electrophysiology

## Abstract

In this study we present a highly versatile and easily configurable system for measuring plant electrophysiological parameters and ionic flow rates, connected to a computer-controlled highly accurate positioning device. The modular software used allows easy customizable configurations for the measurement of electrophysiological parameters. Both the operational tests and the experiments already performed have been fully successful and rendered a low noise and highly stable signal. Assembly, programming and configuration examples are discussed.

The system is a powerful technique that not only gives precise measuring of plant electrophysiological status, but also allows easy development of ad hoc configurations that are not constrained to plant studies.

• We developed a highly modular system for electrophysiology measurements that can be used either in organs or cells and performs either steady or dynamic intra- and extracellular measurements that takes advantage of the easiness of visual object-oriented programming.
• High precision accuracy in data acquisition under electrical noisy environments that allows it to run even in a laboratory close to electrical equipment that produce electrical noise.
• The system makes an improvement of the currently used systems for monitoring and controlling high precision measurements and micromanipulation systems providing an open and customizable environment for multiple experimental needs.

## Graphical abstract

**Figure:**
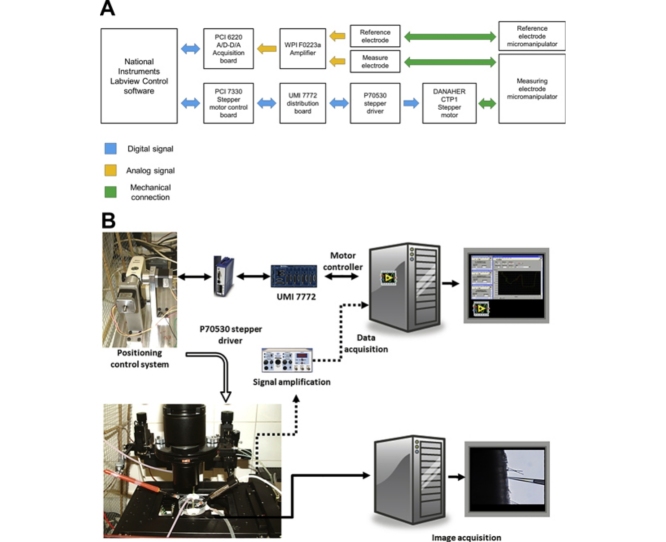

## Method

### System development and assembly

The system consists of different modules: mechanical, electrical, optical and digital. The control center is programmed using Labview object-oriented programming software (National Instruments, USA) to control both the signal acquisition and the micromanipulator movement.

The mechanical part of the system consists of an inverted microscope (Nikon Eclipse TE2000), a couple of positioning micromanipulators (Narishige MM-89, Nikon/Narishige, Japan); one holds the reference electrode and the other holds the measuring electrode The second micromanipulator is coupled to a hydraulically driven high precision micromanipulator (Nikon/Narishige MO-188). The knobs of this micromanipulator corresponding to the x and y axis are coupled to a custom-made retainer that is fixed to the axis of a stepper motor (DANAHER CTP12ELF10MMA00, Danaher Motions, Washington DC, USA) which is electronically controlled by a P70530 stepper driver, connected to a UMI 772 distribution board which in turn is connected to a PCI 7330 dual axis motion controller (all from National Instruments, USA). Detailed characteristics of the steeper motors can be found in the Additional Information section.

The signal acquisition system consists in a WPI FO223a amplifier (World Precision Instruments, London, UK) connected to a PCI 6220 A/D-D/A acquisition board (National Instruments, USA). Both the PCI7330 and PCI 6220 are plugged to a PC computer running windows. Different combinations of operating system and Labview software can be used although in our first development the system was running under Windows XP second edition and Labview 8.6, but we have made successful tests under windows 7 and Labview 11, which shows both the adaptability and compatibility of the system. An exhaustive list of operating system and Labview versions can be seen in Table 1.

**Fig. 1:**
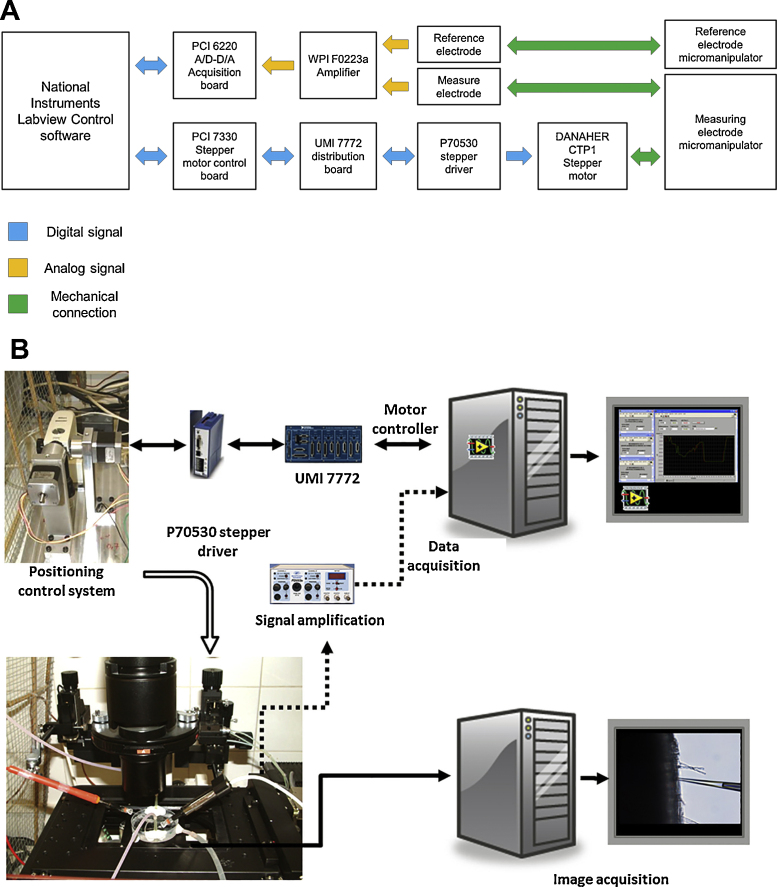
(A) Scheme of the connections needed for the full system (i.e. arranged for measuring ion fluxes). Different colors mean different kinds of connections as explained in the figure. (B) Scheme of the components used and their interrelationships.

A schematic representation of all the connections is displayed in Fig. 1 (A and B) and a detailed photography of the actual sample mounting can be seen in Fig. 7A.

### System programming

**Fig. 2:**
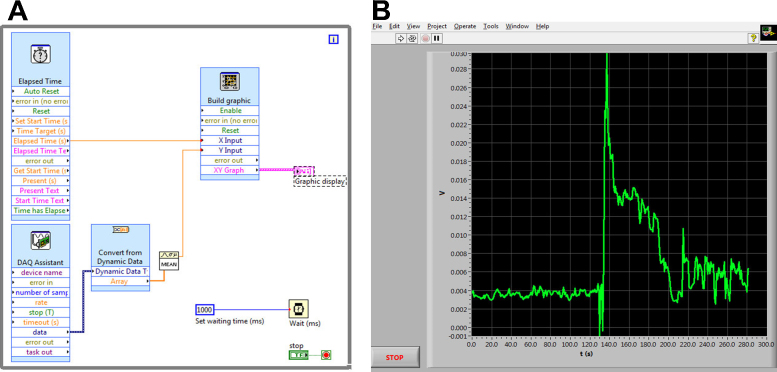
(A) Nuclear programming of the system. This is the simplest configuration and can manage signals that are displayed (B). No data recording is possible in this configuration.

**Fig. 3:**
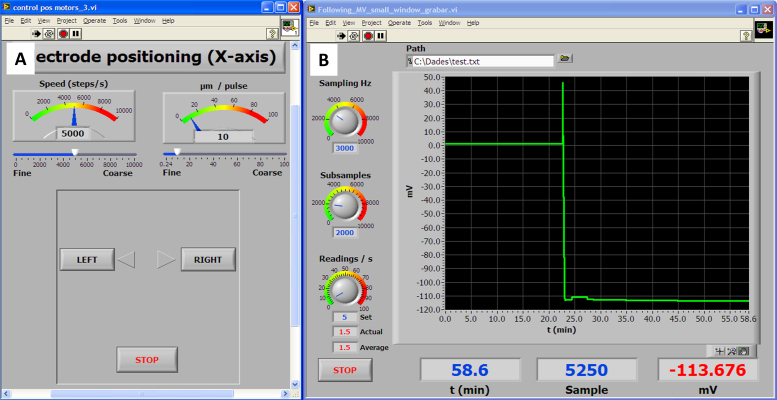
Combination of Virtual panels used for measuring membrane potentials. (A) Control panel for the microelectrode positioning. Two sliders control the movement of the microelectrode. The left one controls the speed at which the tip moves and the right one controls the length of the tip movement. Both are used in combination. (B) Recording VI. Unlike the simple structure shown in Fig. 2, this VI is able to record data and also a combination of controls for sampling frequency, numbers of samples taken to obtain each data point (subsamples) and the intended data readings per second have been implemented. There are also some information indicators such as the actual sampling rate, time, number of samples taken and potential readout.

**Fig. 4:**
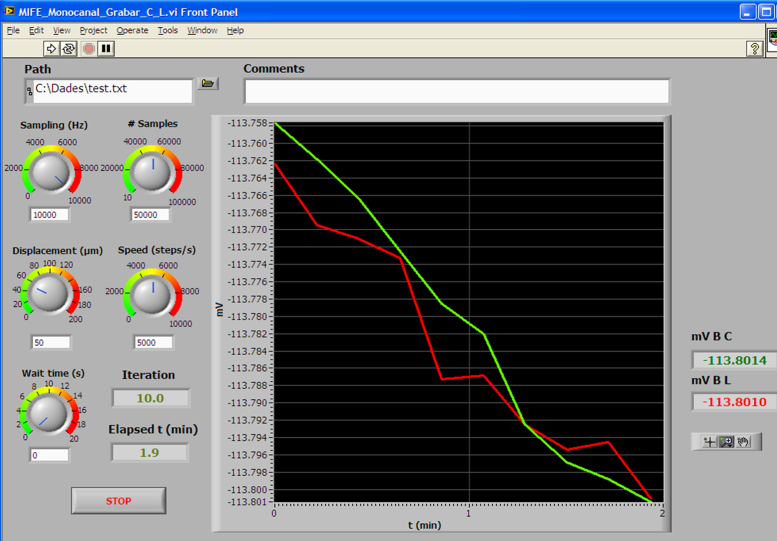
Panel of the Virtual Instrument used for measuring potentials in the ion fluxes measurement configuration. At the left side there are controllers for tip displacement, movement speed and electrode stabilization interval (Wait time). A window for including comments concerning the experiment has been added.

The system was originally programmed using National Instruments Labview 8.6 software running on a Windows XP operating system, but this is not the only possible working environment, since, as stated above, it can run under several combinations of operating systems and Labview versions (for example. Windows 7 and Labview 11). The core of the programming can be seen in Fig. 2 and consists in a Virtual instrument (VI) featuring a very simple loop. Each iteration extracts a number of samples and by averaging them to reduce spurious noise a smooth signal acquisition is achieved. Each value of the average is sent to the graph display together with the timestamp provided by a time counter and a x-y graphic is displayed in which x-axis corresponds to time and y-axis to the intensity of the signal, measured in mV. From this simple nuclear program, a series of different implementations have been done, each one corresponding to a specific need. The two main configurations can be seen in Fig. 3, Fig. 4. Fig. 3B corresponds to a continuous single channel current measuring (for example membrane potential) and Fig. 4 corresponds to ion fluxes measurement configuration.

One of the most important considerations when designing the acquisition software was to take into account that the data could be saved for further calculations and processing. Thus, a data saving module was implemented in a routine that runs before and together with the acquisition process. This routine creates an ASCII tabulator separated text file that can be easily transferred to a Microsoft Excel^®^ file just by dragging and dropping the text file into it. The data files can as well be managed in Mathlab^®^ or other calculation and statistic packages.

### Method validation

#### Noise measurement and performance evaluation of the system

Prior to measurements, the system had to be checked both for mechanical and electrical precision, stability and performance.

The mechanical benchmark yielded a precision of positioning up to of 1 μm with a very high repeatability (less than 0.5% error) using the combination of the hydraulic micromanipulator and stepper motors as listed in the above sections. An exhaustive list of the stepper motors characteristics can be seen in Table 2.

The signal benchmark was conducted doing an empty measurement of voltage in standby mode in order to check the noise discrimination and stability of the acquisition board. From the preliminary data obtained from a set of tests revealed that three issues had to be taken into account.

1. The presence of parasitic noise resulting from earth lines. This issue was solved by plugging the system into two different Uninterrupted Power Supply (UPS) devices. Each UPS unit was plugged into a different power line. The acquisition board and the voltage amplifier were connected to the same UPS while the computer, the stepper motor controller and the stepper motor power supply were connected to a different UPS, thus isolating the earth lines from each other.
2. The power line regional configuration. This issue is related to the coupling of the AC electrical phase with the acquisition frequency of the board. Both frequencies have to be in phase to ensure that each measurement or set of measurements are taken at the same level of the sinusoidal wave of the main power voltage to prevent the formation of a parasitic wave pattern. In our case, the best combination, as suggested by the hardware and software manufacturer, was to fix the board frequency to a multiplier of the mains frequency (i.e. 50 Hz in continental Europe).
3. The third issue was due to the presence of several spikes in the recorded signal generated by devices connected to the general power line of the laboratory (e.g. freezers, stirrers, etc.). It had to be removed by adjusting both the working frequency of the board and the number of samples taken in each cycle to avoid coupling with the parasitic currents generated by the laboratory hardware, that revealed quasi cyclic. In our case, the best combination was a cycling of 3000 samples per second and 200 samples per cycle, which rendered a sampling capacity of 1.5 samples per second. Although this sampling speed may seem rather low for determinate purposes it is fast enough for most measurements. Moreover, this is not the only combination that can be achieved and several other combinations yielded a faster and equally stable reading (circa 20 readings s^−1^). It is worth to remark that it is important to select a good compromise between performance and number of data acquired when developing a new application in order to reduce the number of data without losing accuracy. In fact the acquisition card is not actually slow; it can handle frequencies up to 10,000 Hz. However, working at such high rates, the signal appears with much noise. It is more desirable to work at high frequencies and to average a determinate number of raw data such as 500 or 1000 samples to try to cancel random fluctuations This strategy yields a speed of 20–10 measures per second, allowing a time resolution of 0.05–0.1 s. Any other combination of frequency and averaging can yield better signal-to-noise ratio maintaining the high sampling rates. Since we are working with glass electrodes and not with metallic ones, this speed is good enough for our purposes and limits the quantity of data written in the files, and concomitantly decreases the need of having to process a huge amount of data generated in long-term experiments. With an accurate selection of frequency, averaging and isolation, this device can handle a temporal resolution up to 0.01 s. Of course, using faster AD/DA cards with higher resolution, faster acquisition rates could be achieved, but this will lead to the neurophysiology ranges rather than the plant electrophysiology which is our concern.

**Fig. 5:**
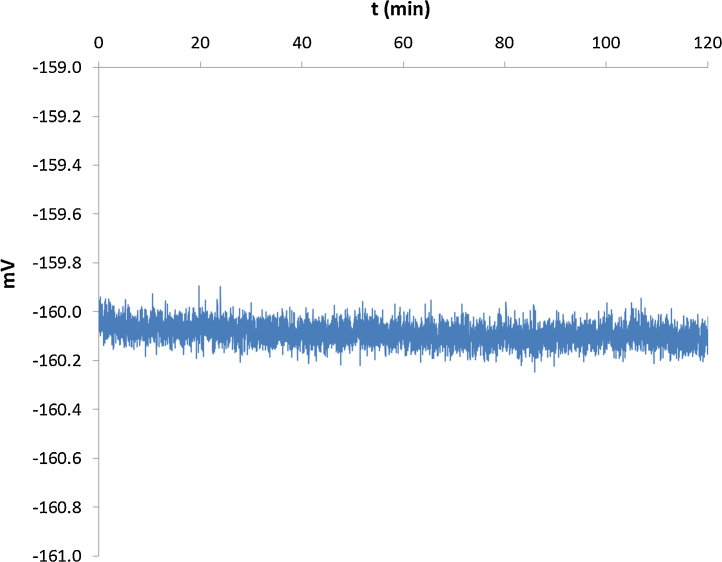
Noise monitoring. A microelectrode loaded with K^+^ selective ionophore was submerged in a solution containing 500 μM K^+^ and, after a stable signal was achieved, it was left to measure during 120 min at a frequency of 300 samples s^−1^ and an averaging of 200 samples, Tre results showed an average voltage of −160.09 ± 0.04 mV and a drift of 20 μV h^−1^ (see Table 3).

**Fig. 6:**
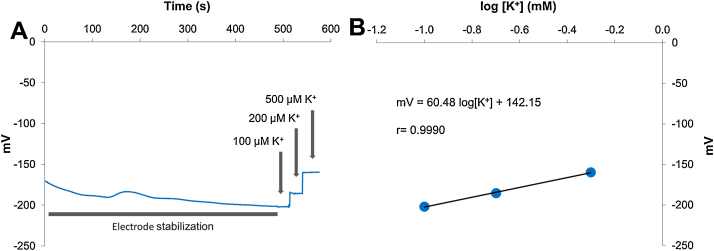
(A) Typical recording of a K^+^ calibration. Spikes due to solution manipulation have been removed. (B) Regression obtained from the potential values obtained in (A).

After adjusting the measuring values, the system was checked under load to test both the stability of the signal (Fig. 5 and Table 3) and the response to a calibration. An example of calibration using K^+^ can be seen in Fig. 6. In our measurements, only calibrations with a regression coefficient equal or higher than 0.999 and a slope of 58 ± 5 were used; otherwise electrodes were discarded.

## Examples of applications

The two main configurations used by our system are continuous direct voltage monitoring, and Ionic flow estimation. The two main configurations are described below and can be summarized as follows:

- Continuous direct measurement (mainly for membrane potential measurements). An example of this procedure can be seen in next section. As a result that only data acquisition is involved, the signal acquisition rate can be very fast as described above. This method is suitable for measuring changes in membrane polarization which often appear within short time after treatment has been applied. Anyway, it has to be taken into account that when changing the bathing solution a disturbance is produced. This is a common issue that can be treated in different ways but the discussion is far away from the purposes of this publication. This disturbance, as can be seen in Fig. 8, can reduce the ability of any system to detect very early changes.
- Vibrating mode. In this mode, readings are taken at two positions in order to measure de gradient generated by the ionic flux across the membrane. Perhaps the word “vibrating” can induce to confusion due to the fact that the common idea of vibration corresponds to a fast change of the tip position which is not the case of ion flux measurements. Unfortunately, in this mode measurements cannot be as fast as in the precedent configuration since the electrode has to reach a steady state, which takes a while; but also the traveling of the tip between the two measuring positions takes its own time to be done. In this case, as in any vibrating electrode, the speed of the readings is considerably reduced if compared with the above configuration. One possible solution could be using two electrodes put at the far and near positions and measuring them at the same time. In this case, each electrode has to be calibrated independently and probably with this system faster measurements could be achieved leading to a further improvement of the technique.

### Membrane potential measurements

**Fig. 7:**
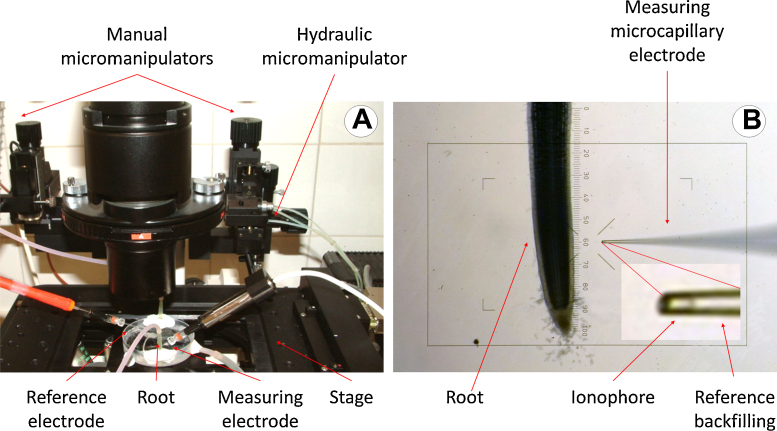
(A) Photograph of the setup used for measuring potential showing the measuring cell, the microcapillaries, the micromanipulators and the inverted microscope stage. (B) Photograph of the arrangement of the elements used for measuring ion fluxes. Insert shows an enlargement of the microcapillary tip with the ionophore loaded.

The system configuration for membrane potential measurements is a combination of x-axis movement and single channel acquisition voltage. As a measuring electrode, a microcapillary filled with 0.2 M KCl with a tip 1–3 μm in diameter was used. As reference electrode, a broad tipped capillary filled with 0.2 M KCl in phytoagar (Duchefa Biochemie BV, The Netherlands) was used (Fig. 7A, left).

Wheat seedlings germinated on phytoagar (0.8%) were transferred to a growing solution (0.5 mM KCl + 0.4 mM CaCl_2_, pH = 6.8 under aeration). After 3 days, seedlings were fixed with BluTack^®^ to a Petri dish cover (5.5 cm diameter) and a bathing solution with the same composition as the growing solution was added. The mounting was put on an inverted microscope stage and the electrodes were immersed in the bathing solution as seen in Fig. 7A. The signal was allowed to stabilize for circa 30 min and once the voltage was stabilized it was corrected to 0. Immediately the microcapillary tip was set in place near the root surface and manually inserted into a cortical cell by using a coarse micromanipulator. When the insertion was successful a fast drop of the voltage was observed and after sealing of the membrane around the microelectrode tip, a stable potential could be attained. This voltage was then monitored.If a leakage was observed (i.e. a drifting of the signal), a fine tuning of the positioning was done by using the stepper motors and moving the electrode in small steps of around 1 μm using the control panel displayed in Fig. 3A until the potential was stabilized again revealing a tight sealing of the membrane. After allowing the system to stabilize for a few minutes, a test substance known to modify the membrane potential was added to the solution with an automatic pipette, first removing the bathing solution and then adding the same solution containing the test substance.

**Fig. 8:**
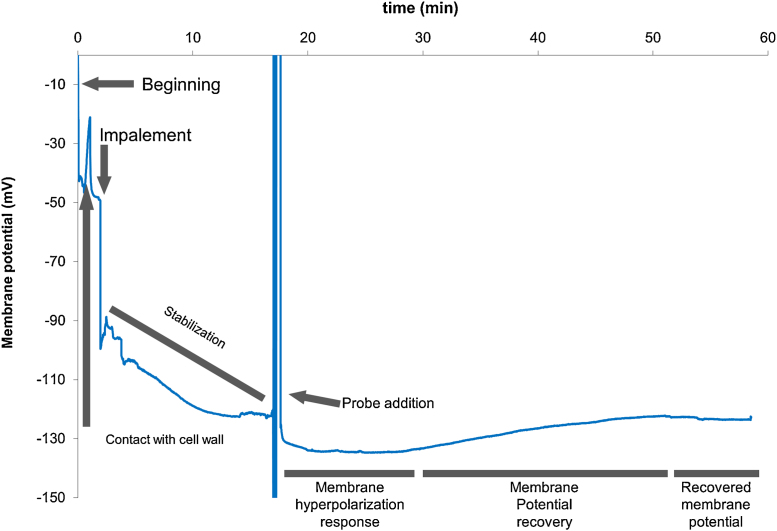
Recording of a membrane potential measurement experiment in which the different effects of the process are explained. This recording depicts clearly a membrane depolarization.

A recording of one of these experiments is shown in Fig. 8, in which all the phases of a typical experiment can be seen. It is remarkable that when changing the bathing solution, a big jump of the measured potential can be observed. This is due both to a mechanical effect caused by the rapid flow of the solution around the root during the change process and to the electrochemical changes due to the withdrawal and release of a new solution around the root surface. Finally, a typical hyperpolarization of the membrane can be observed as an effect of the addition of the test substance followed by a recovery of the initial potential.

In this experiment, two remarkable facts can be taken into account: the high signal-to-noise ratio and the possibility of fine adjustment of the tip position by the use of the stepper motor. This highly improves the impalement success, thus yielding a high rate of valid experiments in a shorter time than if the whole process has to be done only by hand adjusting of the microelectrode tip.

### Ion fluxes measurement (vibrating mode)

This configuration corresponds to the vibrating mode as described above.

**Fig. 9:**
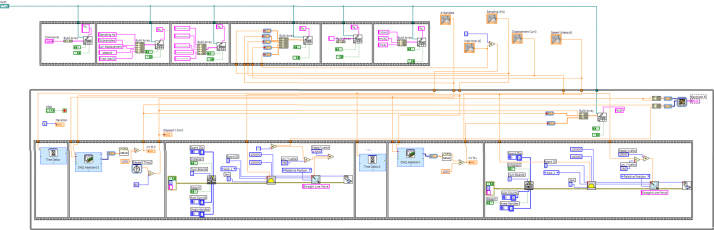
Programming of the cinematic sequence for controlling the movement of the microcapillary and acquisition and recording of voltage at each position. Detailed explanation in the text and Fig. 10.

When measuring ion fluxes, the microelectrode has to be moved to two positions at a different distance of the sample surface in order to get the ion gradient generated by the ion flow due to the release or absorption of ions across the plasmalemma of the cell or organ surface. In this case, a combination of data acquisition and movement of the electrode has been adopted. The Virtual Instrument consists of a cinematic sequence in which each frame controls a subset of actions as shown in Fig. 9. In brief, the system was previously calibrated as described in the section “Noise measurement and performance evaluation of the system” and then the root was mounted as described in the above section and Fig. 7A. However this time the microcapillary tip had been filled with a suitable ionophore (see detail in Fig. 7B) and was not inserted into any cell. At the beginning of the measurement the parameters of the program are sent to a file and afterwards the sequence is started. To avoid accidents, at the beginning of the experiment the microelectrode is always positioned to the closest distance to the surface as seen in Fig. 7B. According to the sequence displayed in Fig. 9, the first movement brings the microelectrode to the far position. For each cycle, the first frame takes the measure of the voltage after allowing the electrode to be conditioned for several seconds to ensure that equilibrium has been achieved. After doing this, the time and the voltage are sent to the text file and recorded, and the second frame begins to run. In this part of the sequence, the motor is turned a determined number of steps moving the micromanipulator a known distance backwards to the far position. After allowing again to stabilize, a new set of data is taken and sent to the text file. Once the measurement is achieved and saved, the cycle begins again by regaining the near position, continuing until the end of the experiment.

The interaction between the Labview cinematic module and the micromanipulator is as follows: the cinematic module controls the instructions that are sent to the PCI 7330 Stepper motor controller board. This board sends a signal to the UMI 7772, that is an external signal distributor and the separate signals from the two different axes are sent to a PT0530 stepper driver that controls the motor movement. All this process is automatically done by the codes sent from the PCI 7330 unit once programmed in the corresponding Labview Virtual Instrument. Since the knobs of the micromanipulator are attached to the retainer, which in turn is fixed to the stepper motor, each movement of the motor makes the knob turn a determined number of degrees and move the plunger that exerts or withdraws a pressure to the oil that fills the micromanipulator. This variation in pressure is transmitted to the positioner attached to the microscope, resulting in a forward or backward movement of the microcapillary.

The precision achieved by this combination of measuring and movement is of 1 μm displacement and 0.1 mV potential.

**Fig. 10:**
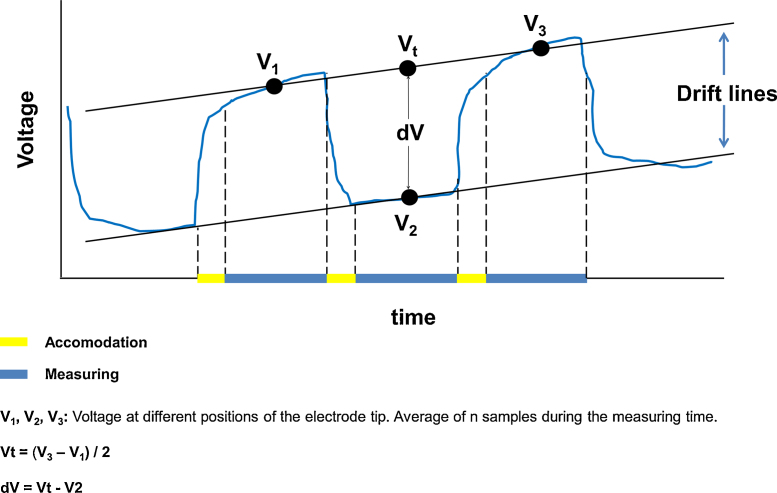
Scheme of the process for obtaining the potentials in the ion flux measurement configuration. The yellow bars, corresponding to the accommodation time are controlled by knob “Wait time” in Fig. 8 and the process of measuring and averaging are controlled by the knob "# of samples” in Fig. 4. The data sent to the text file are the values VI, V2, V3, … which correspond to the average of the values obtained during the blue bar period of time. The use of 3 points to determine *d*V minimizes the effect of the drift of the measure that usually appears when taking measures. Modified from Newman et al. [2].

For the calculation of ion fluxes, several considerations based on the Nernst’s equation have to be taken into account as described in the next sections and displayed in Fig. 10.

## Calculations

Although measurement of single potentials are directly conducted by the system, the ion fluxes need more complicated calculations because different factors like the geometry of the organ affect the gradient generated by the surrounding ionic flux. Since we are measuring a concentration gradient other factors have to be taken into account (i.e. the distance from the near and far positions or the geometry, shape and diameter of the microcapillary tip).

Ion flux is calculated using calibration results [1] and root geometry as shown in the following equations [2].

*J* *=* *c u F* (*58/Nernst slope*)(d*V/*d*x*)

d*x* *=* *r*^2^ [1/(*r* + *x*) − 1/(*r* + *x* + d*x*)], for the sphere

d*x* = *r* ln[(*r* + *x* + d*x*)/(*r* + *x*)], for the cylinder

where

*J*: Net flux of the ion (mol m^−2^ s^−1^).

*c*: Ion's free concentration (mol m^−3^).

*u*: Mobility of the ion (m s^−1^ N mol^−1^).

*F*: Faraday's constant (96,500C mol^−1^).

*Nernst slope*: Slope determined by the calibration procedure.

d*V*: Electric potential difference between the two positions of the microelectrode (volts).

*x*: Distance of the tip at the closest position (m).

d*x*: Distance between the two measuring positions (m).

*r:* Radius of the measured object (m).

In our case, we adopted a similar procedure as in the Woods Hole system [3], [4] in which, instead of storing the raw voltage data, only the average voltage of each frame is recorded in order to get the minimum number of data while keeping the measuring accuracy. This simplifies the programming of the system and allows an easy way of calculating dV with a spreadsheet without involving complicated programming of the acquisition software. In Fig. 10 an example of the procedure can be seen.

For calculating the dV at each interval, a modification of the method from Newman [2] has been applied. Instead of using the slope of each data set, the mean voltage is sent to the text file as explained below. First, for each measuring position, the electrode is allowed to stabilize for a determined period of time chosen individually from its characteristics (yellow bars). This permits to omit data points occurring during electrode settling after movement [2]. After this period, the measuring starts and the system takes a determined number of samples and outputs the average voltage (V1, first blue bar). Then, the electrode is displaced to the next position and allowed again to stabilize (second yellow bar). A new set of samples is taken and a new average voltage is output (V2, second blue bar). The cycle continues until the experiment is finished (V3 and further measurements).

In this configuration, only the first positioning of the microelectrode tip and the bathing solution change has to be done by hand, while the rest of the experiment is automatically conducted by the system’s program which is displayed in Fig. 9.

Unlike in the previous experiment, the concentration of a determined ion is being measured by using a specific ionophore loaded into the microcapillary tip (insert in Fig. 7B). For this purpose it is necessary to calibrate the electrode before the beginning of the experiment, because although electrodes should follow Nernst’s equation, which defines the electrical behavior of an ion in a determined electrical cell, there are always slight differences between different sets. Calibration was conducted by submerging the electrode, previously filled with the reference solution and the suitable ionophore at the tip, into solutions with known concentrations of the ion to be measured (in this example, KCl). A typical calibration process can be seen in Fig. 6A, B.

**Fig. 11:**
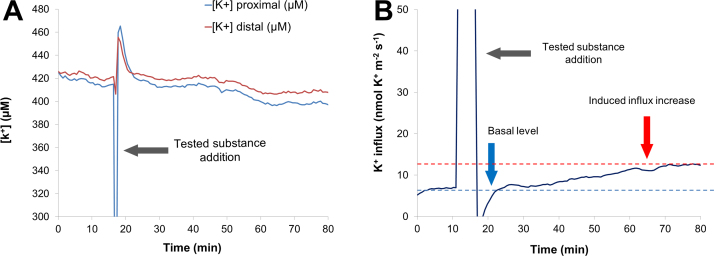
(A) Ion flux measurement experiment recording. The concentrations of the measured ion at both near and far positions of the microelectrode tip are displayed. A bigger difference in ion concentration among both positions implies a higher flux. (B) A processed flow estimation taken from (A). It can be seen that the broadening of the difference in ion concentration seen in (A) is turned in a higher ion flux. In this case, influx.

Once the electrode was set in place near the root surface (typically about 30 μm), the measuring program was started as described in the methods section. Fig. 11 shows the recording of a typical experiment using the same substance used to check the membrane potential measurement. Again, a jump can be seen when changing the solution.

In this case, it can be seen that the K^+^ concentration is higher at the distal than at the proximal position of the microelectrode tip (Fig. 11A), indicating that a K^+^ influx is being conducted across cell membrane. After the flux recovered, the effect of the test substance on the K^+^ flux is expressed by an increase of the influx seen by the fact that the gap between proximal and distal K^+^ concentrations is being broadened (Fig. 11A). The result of the calculations are shown in Fig. 11B. The combination of the two example experiments shown in these two sections clearly depicts that the test substance has caused an early change in membrane properties that finally affected K^+^ flow across membrane.

## Advantages of the method

• Possibility of control electrophysiological data acquisition with a high precision of microelectrode positioning, both at the intra- and extracellular level.
• The described automation of the system allows the operator to be outside the measurement room during the data acquisition process, thus lowering possible sources of noise, both electrical and mechanical-.
• High modularity due to the use of Labview programming environment. The selected combination of microscope and micromanipulators are not unique and other kinds of microscopes and micromanipulators can be used since they are independent from the programming.
• High precision thanks to the use of hydraulically oil-driven micromanipulators directly connected to stepper motors.
• High signal-to-noise ratio due to the fact that the power line of the stepper motors and the current amplification lines are connected to different isolated UPS systems.
• Capacity of achieve a high precision accuracy in data acquisition under electrical noisy environments even without a Faraday’s cage. This allows to run the electrophysiological system even in a laboratory close to different electrical equipment such as deep freezers, refrigerated growth chambers, magnetic stirrers, etc. that produce electrical noise.
• The system designed here makes an improvement of the currently used systems and allows an easy, highly configurable and expandable system for monitoring and controlling high precision measurements and micromanipulation systems providing an open environment that can be customized for multiple experimental needs.
• The use of the described kind of control creates a smooth movement that allows a very low disturbance of the unstirred layers surrounding the root or cell surfaces. When penetration of the electrode into the cell is required, the system assures minimal injury to the plasma membrane.

## Additional information

### Fundamentals of electrophysiological measurements in plants

Cell walls and membranes are the primary sites of interaction of many substances with plants [5], [6]. The cell plasma membrane is a highly regulated system that controls the permeability and transport of ions and many other substances in and out of the cell. Many different transport systems regulate this traffic across the membrane. Available soil chemicals can interact with the plasmalemma and modify its properties [7]. Early consequences of these modifications are changes in the transmembranal ion transport, alterations of signal transduction pathways and even toxicity [8]. Since both ionic fluxes and the electrochemical status of the membrane are markers of the effects of many of these substances, specific measurement techniques have been developed that currently are widely used in mechanistic studies not only concerning plant nutrition, but also signaling processes and abiotic stress responses.

The uptake and transport of mineral nutrient ions by plant roots has been studied for many years using different methods of chemical analysis and tracer techniques [8]. The development of new techniques such as membrane potential measurements, patch clamp, ion-and pH-sensitive dyes with advanced imaging techniques, and ion-selective microelectrodes has provided specific information on membrane transporters and ion distribution and movement. These techniques include both invasive procedures such as membrane potential measurement and non-invasive measurements of ion fluxes using ion-selective microelectrodes. Both types of techniques have contributed to the functional characterization of the transporter systems.

Ionic fluxes can alter the ionic status of the root-surrounding medium due to the movement of charged particles. Potentiometric techniques can be used to measure these alterations. The original idea of using electrochemical potentials to determine ion concentration outside plant tissues to calculate ion fluxes was given by Lucas and Kochian [8] as consequence of applying Nernst's equation to mineral nutrition, which was first applied by Newman et al. in the measurement of H^+^ and K^+^ fluxes in corn roots [9]. In this case, the main technique consists of the measurement of the ionic gradient generated by the ions moving out of or into the root by positioning a selective electrode in different positions into the unstirred layer near the root or cell surface in order to measure the intensity of this flow. This is the so called Microelectrode Ion Flux Estimation technique (MIFE) [9], [10], [11].

Further development of the technique has been applied in other laboratories and the range of ions has increased with the development of more selective ionophores being of special relevance to the group of biophysics at the University of Tasmania. Several systems with different degrees of automation have been developed in different countries [10], [11], [12], [13], [14], [15], [16], [17], [18], [19], [20].

The system is not limited to ion studies, but also oxidoreduction reactions can be used to determine other nonionic molecules such as auxin [21]. Unfortunately, due to the small size of the measurement areas, microelectrodes must be used. This implies that the involved currents and intensities are very low. Thus, precise and powerful amplification devices have been developed. But the intensity of the current is not the unique issue in measuring electrophysiological effects and hence, a precise control of the spatial positioning of the electrode tips is also required.

Although excellent commercial microscopes and micromanipulation devices have been developed, they are mainly intended for steady measurements and usually there are no manufactured instruments devoted to measurements with vibrating electrodes. Therefore, it is necessary to create them by customizing modified commercial devices. In addition, usually *ad hoc* specific programming is needed to control and manage the signals generated by these systems and only a few laboratories have developed and constructed these kinds of assemblies. For a good compendium of the different electrophysiological techniques in plants, refer to Volkov [22].

### Considerations on noise sources

This is a topic of much concern among researches working with such small intensities as used in electrophysiological studies that range in the dominion of nA. Quoting the excellent manual from Skoog et al. [23]: “Unfortunately, noise-free data […] can never be realized in the laboratory because some types of noise arise from thermodynamic and quantum effects that are impossible to avoid in a measurement. In most measurements, the average strength of the noise N is constant and independent of the magnitude of the signal S. Thus, the effect of noise on the relative error of a measurement becomes greater and greater as the quantity being measured decreases in magnitude”.

But not only are these physical effects responsible for the appearance of electrical noise. The main sources of noise are the thermal noise, shot noise, flicker noise and environmental noise. The three first ones are inherent to any physical system and related to thermodynamics and the quantic structure of matter, but the last one is directly caused by nature (electrical storms, solar EM radiation, local variations of magnetic field, etc.) and from anthropic origin (power lines, TV and radio sources, relay triggering, electric motors, action of lighting switches, RF from computers and cellular phones, etc.). Since any conductor is a potential antenna capable of picking up EM radiation and convert it into an electrical signal, good care has to be taken to prevent parasitic noise, such as shortening electrical connections, shielding any conducting wires and isolating earth lines as much as possible. One of the most used implementations for EMI is the Faraday’s cage [24], [25] but some other solutions can be used, such using an *in situ* data logger that can send data after conducting the experiment [25]. Our experimental implementation makes it not possible to use the last mentioned solution and since in our laboratory, we have many of the above described EM sources and at the early stages of development of the device we experienced considerable problems with signal stability and spurious peak production. Those could not be related with the electrode responses since the frequency was random and not related with the electrode response speed. Then, as is indicated above, we realized two facts: the first is that taking a slower data speed acquisition the spikes dramatically decreased, and the second was that isolating the power source of the device from the main power source from the laboratory by installing UPS units, one dedicated to the steppers feed and a second one dedicated to the electrophysiological devices, not only the spikes disappeared, but also the amplitude of the noise was also smaller. So we removed the Faraday’s cage and the acquisition was kept stable and with low noise.

## Competing interests

This work was supported by the Spanish MICINN (Projects BFU2013-42839-R).

Ana Rodrigo-Moreno. PhD fellowship from Ministerio de Ciencia e Innovación (BES-2008–005096).

Simone Rankl stage in Barcelona has been funded by COST Action FA1103.

## Author’s contributions

Benet Gunsé has developed, programmed and tested the system and is in charge of its maintenance and evolution. Charlotte Poschenrieder and Juan Barceló introduced the idea of constructing this system in the framework of the projects that are carried out in the laboratory. Ana Rodrigo-Moreno has implemented the ion fluxes measurement technique and made her PhD thesis under supervision of Dr. Poschenrieder and Dr. Gunsé by using this installation among different other techniques. Simone Rankl used the system during her two stages in our lab and introduced the use of membrane potential measurements that are being carried out with the system described here under supervision of Dr. Poschenrieder and. Dr. Gunsé and under the direction of Dr. Peter Schroeder.

## Figures and Tables

**Table 1:** Labview versions and its compatibility with Windows operating systems. Taken from http://digital.ni.com/public.nsf/allkb/B972242574D4BB99862575A7007520CBhttp://digital.ni.com/public.nsf/allkb/B972242574D4BB99862575A7007520CB

**Table 2:** Stepper motors characteristics.

NEMA 17 Motor (CTP12ELF10MMA00)	
Electrical	
Step angle	1.8°
Steps per revolution	200
Angular accuracy	±3%
Phases	2
Current per phase	1.0 amps/phase
Industry standards	
Industrial standards	CE, UR
Sealing standards	IP40
RoHS compliance	Yes
Physical	
Operating temperature	−20 to 40 °C
Shaft load (20,000 h at 1500 rpm)	15 lb (6.8 kg) at shaft center
Shaft speed	3000 rpm
Radial axial push	2.7 kg
Axial pull	6.8 kg
Recommended heat sink size	10 × 10 × 1/4 in. aluminum plate
Drive	P70530
Amps/Phase	1.0
Holding torque (N. m)	0.30
Rotor inertia (kg-m^−2^ × 10^−3^	0.0040
Phase inductance mH	7.7
Phase resistance Ω ± 10%	5.25
Detent torque (N m)	0.014
Thermal resistance (°C/watt)	6.21
max speed rpm	3000

**Table 3:** Results of the stability test calculated from the data acquired in Fig. 5.

Stability	Average (mV)	−160.09
	σ_n−1_	0.04
Absolute variation	Max (mV)	−159.84
	Min (mV)	−160.31
Drift	mV/min	0.00
	mv/h	−0.02
